# “New Users” Are Confusing Our Counting: Reaching Consensus on How to Measure “Additional Users” of Family Planning

**DOI:** 10.9745/GHSP-D-16-00328

**Published:** 2017-03-24

**Authors:** Aisha Dasgupta, Michelle Weinberger, Ben Bellows, Win Brown

**Affiliations:** a Marie Stopes International, London, UK.; b United Nations Population Division, New York, NY, USA.; c Avenir Health, Washington, DC, USA.; d Population Council, Lusaka, Zambia.; e Bill & Melinda Gates Foundation, Seattle, WA, USA.

## Abstract

FP2020's overarching goal is framed around the new metric of “additional users.” This measure inherently captures population-level change but has been conflated with other ambiguous metrics, such as “new users.” Therefore, we propose a standard set of terms to provide more consistent measurement. Although commonly used service-level metrics cannot be directly compared to the population-level metric of additional users, we describe 2 modeling approaches that can allow service-level data to inform estimates of additional users.

## INTRODUCTION

In July 2012, the London Summit on Family Planning reenergized the reproductive health field by establishing a new commitment to bring modern contraception to women and girls with an unmet need for family planning—those who say they do not want a child soon or at all but are not currently using contraception. At that time, it was estimated that 222 million women in the developing world had an unmet need for modern contraception.^1^ Most of these women were concentrated in the world's 69 poorest countries.^2^ The family planning community committed at the Summit to enabling an *additional* 120 million women in these 69 countries to use modern contraception by 2020.^2–4^ The community felt that designating a single number would help rally the community and push forward a renewed focus on family planning.^3^

Nearly 5 years later, the widely recognized “120 by 20” goal supported by the Family Planning 2020 (FP2020) global partnership can be credited for galvanizing renewed commitment to family planning. However, the new metric of “additional users”—an aggregate metric that estimates how many more modern contraceptive users there are now compared with the estimated 2012 baseline number—has created confusion about the definition and meaning of several other related family planning metrics, including “new users,” “acceptors,” “first-time users,” and “adopters.” It has also raised the question of how service-level metrics collected by programs can be linked to the aggregate concept of “additional users” to assess progress of individual programs toward population changes in contraceptive use at the country level. In this article, which follows from a panel discussion among the 4 coauthors held during the 2016 International Conference on Family Planning, we outline several of the metrics currently used to measure family planning program progress and propose a preferred set of service-level metrics to inform contributions to the FP2020 aggregate-level goal of reaching “additional users.” We also describe 2 approaches—Track20's Family Planning Estimation Tool (FPET) and Marie Stopes International's Impact 2 model—for bridging the gap between service-level measures available in programs' routine service statistics and the aggregate metric of additional users. Finally, we draw attention to the need for more robust data collection systems that allow for the collection of harmonized routine longitudinal metrics rather than focusing solely on visit-based service statistics or cross-sectional household surveys.

## ALIGNING INDICATORS: WHY IT MATTERS

Metrics, especially when used by donors and governments to set goals and measure performance, can drive how family planning programs are designed and implemented. Using the right metrics ensures that program growth translates into additional impact, gives credit for ensuring current family planning clients have continued access to services, and links programmatic increases in contraceptive use with expanded national coverage, not just attracting clients from other providers.

Not all current data management systems enable effective monitoring of family planning program performance. A key dimension to the success of any family planning program that is not fully captured with service statistics, for example, is the effective, voluntary use of a preferred contraceptive method by each program beneficiary over time. Reasons for method-specific discontinuation rates are critical to understanding this longitudinal perspective but this information is often not captured with service statistics. Researchers have instead created models that use available service statistics to estimate program performance.

Several types of indicators are collected to measure family planning program performance, and in particular contraceptive use. Some of these indicators, such as “commodities distributed” and “first-time users,” are collected through routine client-level service statistics or client exit interviews, whereas others, such as “users” and “additional users,” are estimated in models or collected from population-level surveys. To ensure clarity of terminology used throughout this article, we summarize these definitions in the Box.^5^

BOX **Key Terms Used to Talk About Women Using Contraception**

**Commonly Collected Routine Service Statistics Data**

- **Client visits:** The number of times clients interacted with a provider for contraceptive services. In most cases, the same client is counted multiple times because the client comes for multiple visits (e.g., 4 injections over a year). Most health management information systems (HMISs) count client visits.
- **Clients served:** The number of clients who received contraceptive services in a given time period, often 1 year. This is often counted using a client-based HMIS and thus is not very common as few systems have the means to track a uniquely identified client across multiple visits (usually requires an electronic-based system).
- **Commodities distributed/services provided:** The number of contraceptive commodities distributed or services provided to clients (e.g., number of pill cycles, number of IUDs, number of male sterilization services). In some cases, this may be captured at the client level (e.g., counted when products or services are provided to clients), while in other cases they might be counted further back in the supply chain (e.g., counted when products are distributed to a clinic). These counts are often aggregated into the couple-years of protection (CYPs) measure.

**Family Planning Client Characteristics Data Captured Routinely or via Client Surveys**

- **First-time user:** A person who starts using modern contraception for the first time in her life.
- **Lapsed user:** A person who has used modern contraception at any time in the past, but is not currently using a modern method.
- **Adopter:** A client who was not using a modern contraceptive method at the time of her visit, which includes first-time users and lapsed users. The definition of “time of her visit” can vary, for example, today, last month, or last 3 months.
- **Provider-continuer:** A client who, at the time of her visit, was already using a modern contraceptive method that she received from the same service provider (or same network) and comes back for another family planning service (e.g., for resupply of the same method or to switch methods). The definition of “time of her visit” can vary, for example, today, last month, or last 3 months.
- **Provider-changer:** A client who, at the time of her visit, was already using modern contraception and comes for another family planning service, but who had previously received her family planning from a different provider. The definition of “time of her visit” can vary, for example, today, last month, or last 3 months.

Note: The 3 terms adopter, provider-continuer, and provide-changer are mutually exclusive groups: all clients served fall into only 1 of these 3 categories. Collectively, these 3 terms are often referred to as the “client-use profile.”

**Population-Level Data (not directly captured in routine data)**

- **User:** A person who is currently using contraception, regardless of when the method was received. This is not directly comparable with the number of clients served in a year, because it includes women still using long-acting or permanent methods received previously (e.g., a woman who had an IUD inserted in 2012 may still be an IUD user in 2015). This can be estimated through population-based surveys, such as Demographic and Health Surveys, Multiple Indicator Cluster Surveys, or Performance Monitoring and Accountability 2020 surveys, or through modeled estimates of the contraceptive prevalence rate and the number of women of reproductive age (e.g., Track20's Family Planning Estimation Tool and United Nations Population Division estimates) or modeling from service provision data (e.g., Marie Stopes International's Impact 2 model). Note that “currently using” can be interpreted differently by women asked about current use in survey questionnaires.
- **Additional users:** The net number of current contraception users above a specified baseline; in the case of FP2020, the baseline is the number of current contraception users in 2012 in the world's 69 poorest countries. Note that this concept does not apply to an individual but rather to an aggregate population.

***Terms We Suggest Dropping***

- **New user:** A term that has multiple definitions including first-time user, new to the provider (e.g., provider-changer), new to the method (e.g., switching methods), not recently using a method (e.g., lapsed user), and even additional user.
- **Acceptor:** A term that has multiple definitions including first-time user, new to the provider (e.g., provider-changer), new to the method (e.g., switching methods), not recently using a method (e.g., lapsed user), using a method after an abortion or birth, and even additional user.

Because of the ambiguity with the terms “new user” and “acceptor” and because the concepts are adequately captured in other clearer terms, we suggest the family planning community drops these 2 terms from our list of terminology.

## THE AGGREGATE MEASURE OF “ADDITIONAL USERS” BASED ON HOUSEHOLD SURVEYS AND MODEL-BASED ESTIMATES

“Additional users” is inherently an *aggregate* metric indicating how many more modern contraceptive users there are across the 69 poorest countries now compared with the estimated 2012 baseline number of modern contraceptive users in the same countries. Each year, the FP2020 Secretariat publishes its annual progress report showing progress toward the “120 by 20” goal—FP2020's first core indicator.^6^^,^^7^ The additional user results are shown at the country level as well as summed across all 69 countries. At the country level, the number of additional users is calculated using 2 variables, the estimated modern contraceptive prevalence rate (mCPR) and the estimated number of women of reproductive age (WRA), at 2 time periods, currently and the 2012 baseline, as follows:

$$\begin{array}{c} Additional\;users=(WRA_{YYYY}\times mCPR_{YYYY})-(WRA_{2012}\times mCPR_{2012})\\ Where\;YYYY=the\;current\;time\;period\;of\;interest \end{array}$$

The estimate of current mCPR (FP2020's second Core Indicator) is a model-based estimate, informed by nationally representative household surveys, such as the Demographic and Health Surveys (DHS), Multiple Indicator Cluster Surveys (MICS), and Performance Monitoring and Accountability 2020 (PMA2020) surveys, as well as service statistics (in select countries) and historic regional and global patterns of change.

For FP2020, the goal of reaching 120 million additional users can only be achieved if family planning programs (1) continue to sustain services to more than 270 million women,^8^ the number already using modern contraceptives in the world's 69 poorest countries when the FP2020 initiative began in 2012, and (2) further grow the number of users beyond this base. Thus, priority must be placed both on reaching non-users and on ensuring women who are currently using contraceptives have continued access to high-quality services to minimize discontinuation due to dissatisfaction.^9^

To date, national and global-level understanding of progress in family planning has typically been informed by household surveys, including DHS, MICS, and PMA2020, as well as other national and cross-national survey programs (such as the Contraceptive Prevalence Surveys, Reproductive Health Surveys, and World Fertility Surveys). Such surveys are invaluable in providing a cross-sectional insight into contraceptive use, typically from questions such as “Are you currently doing something or using any method to delay or avoid getting pregnant?” However, surveys have limited ability to capture the dynamic longitudinal nature of contraceptive use, including first-time use, discontinuation, switching of methods, resumption of use, and so on. An exception is the calendar section of the DHS questionnaire, which captures a woman's retrospective self-reported contraceptive status (and method), pregnancies, births, breastfeeding status, and method terminations every calendar month for the 5 years prior to interview. Although these data do not suffer from problems of loss to follow-up, the calendar data are vulnerable to selection bias as only women surviving to interview can report, and there are likely to also be memory recall issues. Besides DHS's calendar method, there are some recent examples of electronic client information systems^10^ and a handful of specialist studies^11^^,^^12^ that have captured method switching or discontinuation, but overall conventional measurement approaches to contraceptive use are unable to capture this type of detail.

## LINKING INDIVIDUAL CLIENT AND VISIT DATA FROM ROUTINE SERVICE STATISTICS WITH THE AGGREGATE MEASURE OF “ADDITIONAL USERS”

Performance monitoring of program outputs and trends in family planning services occurs below the aggregate national and global levels, and presents different measurement challenges. Examples include government monitoring of provision of family planning services through the public sector or a private service delivery organization monitoring provision throughout its delivery network. In either case, program monitoring generally relies on routine data from an HMIS. These data originate from registers kept at family planning service delivery points, in which key programmatic elements are recorded on a daily basis, such as date of visit, age and gender of the client, type of family planning service provided, and so on. Yes, information available from HMIS and other routine systems are notoriously compromised by data quality issues, but they are valuable for indicating basic details of service delivery.

At the same time, programs are increasingly interested in understanding how program-level outputs contribute to population-level changes in contraceptive use in the country. More specifically, they want to know whether their services are contributing to an increase in the number of additional users nationally. To answer this question, one must connect outputs measured at an individual level (e.g., clients served) to an aggregate change in contraceptive use nationally (e.g., additional users). As an attempt to bridge this gap, some programs capture information on recipients of services. The most commonly collected client attributes are “adopters” and “first-time users.” These metrics have their merits and can inform aspects of increasing access to contraception. However, they are not comparable with each other and are not the same as population-level “additional users.”

To build a bridge between service and population-level measures, we first need to select the most appropriate service-level metrics that can help inform a program's contribution to population-level contraceptive use. We then need a way to account for both uptake and discontinuation of contraception as women move in and out of contraceptive use throughout their lifespan.

### Service-Level Metrics: What Do They Tell Us?

At the individual level, a “first-time user” is a woman who initiates contraception having had no previous experience with contraceptive use. A client can be a first-time user only once during her lifetime. In contrast, an “adopter” is someone who starts using family planning who was not currently using modern contraception at the time of her visit, but may have used modern contraception in the past. Thus, a woman could be an adopter several times during her life if she stops and starts using contraception. As adopters include both first-time users and lapsed users, in the context of program-level monitoring, the number of first-time users will always be lower than the number of adopters.

When assessing a program's contribution to increasing contraceptive use nationally, adopters is a more useful metric and preferred over first-time users, since it is a more inclusive measure of adding women into current contraceptive use. Ideally family planning providers or organizations would capture a full suite of indicators including both adopters and first-time users. In reality, they often have to choose a handful of indicators from a longer list to make routine data collection manageable for service providers; hence, the need to prioritize which indicators to include. It is important to note, however, that capturing the number of adopters is not sufficient alone to estimate how national levels of contraceptive use are changing, as will be discussed below.

The terms “new user” and “acceptor” have been frequently used and misused in multiple contexts to refer to a first-time user, an adopter, and even an additional user. In recent years, “new user” has often been used incorrectly to refer to measuring contributions toward FP2020's goal of 120 million additional users. Not only is the “new user” term ambiguous and confusing—“New” to contraception or “new” to the provider? “New” as differentiated from the 2012 baseline of modern method users?—these measures of “new” do not adequately capture important concepts associated with measuring additional users. We suggest, in the interest of clarity, to drop the terms “new user” and “acceptor” from the family planning metrics language and replace them with the clearly defined term of first-time user, adopter, or additional user, as appropriate.

### Service-Level Versus Population-Level Measures: Why They Are Not Directly Comparable

Maintaining the 2012 baseline number of modern contraceptive users in the world's 69 poorest countries does not mean that the same 270 million women will continue to use contraception year after year. Women will move in and out of contraceptive use over time, as their needs and situations change. There are many reasons a woman might discontinue use of contraception, such as ageing out of her reproductive years, mortality, method-related reasons when still in need (including health concerns or side effects, partner disapproval, cost, or access challenges), method failure, or no longer needing contraception due to not being sexually active, wanting to get pregnant, partner separation/dissolution, or menopause.^13^^,^^14^ Population-level estimates of contraceptive use at different points in time capture the fact that there is both uptake and discontinuation of contraception.

**FIGURE 1:**
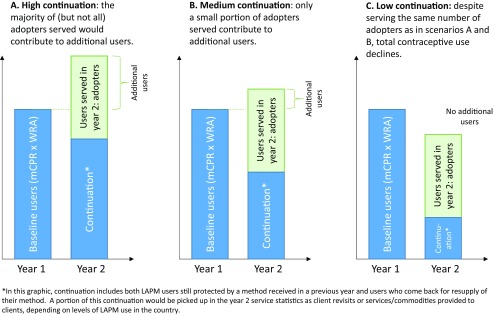
Contribution of Adopters to Additional Users Depending on High, Medium, and Low Continuation Scenarios Abbreviations: LAPM, long-acting and permanent methods; mCPR, modern contraceptive prevalence rate; WRA, women of reproductive age.

However, tracking indicators that are captured at the service level (e.g., number of adopters) focuses on one side of the equation (gains) without measuring the other side (losses), and so does not allow us to see the full dynamic of continuation, discontinuation, and net change. One has to account for levels of continuation and discontinuation to know how many of the adopters are “replacing” women who moved out of current use and how many are “adding” to total current use. Without seeing the full equation, it is not possible to know the extent to which the number of adopters are offsetting declines due to women dropping out from the baseline (Figure 1). In fact, in an extreme case it is possible that despite reaching a large number of adopters, total contraceptive use nationally could stay the same, or even decline. Therefore, program-level data that capture information about individual clients served or client visits are not a direct measure of changes in population-level contraceptive use. Further complicating this issue is that most HMISs are visit-based rather than client-based. Since some methods require clients to make multiple visits over 1 year of use (e.g., injections) while other methods require only 1 visit over several years (e.g., implants), visit numbers must be adjusted to the approximate number of “users.” In some instances, data that refer to individual visits are labeled and interpreted as individual users or clients, creating further confusion.

#### Tools and Models Can Bridge the Gap Between Service and Population Measures

When available, client-level data can be used to inform national level changes in mCPR and estimate contributions by particular organizations to national-level changes using existing tools and models, namely, FPET and the Impact 2 model.

**FIGURE 2:**
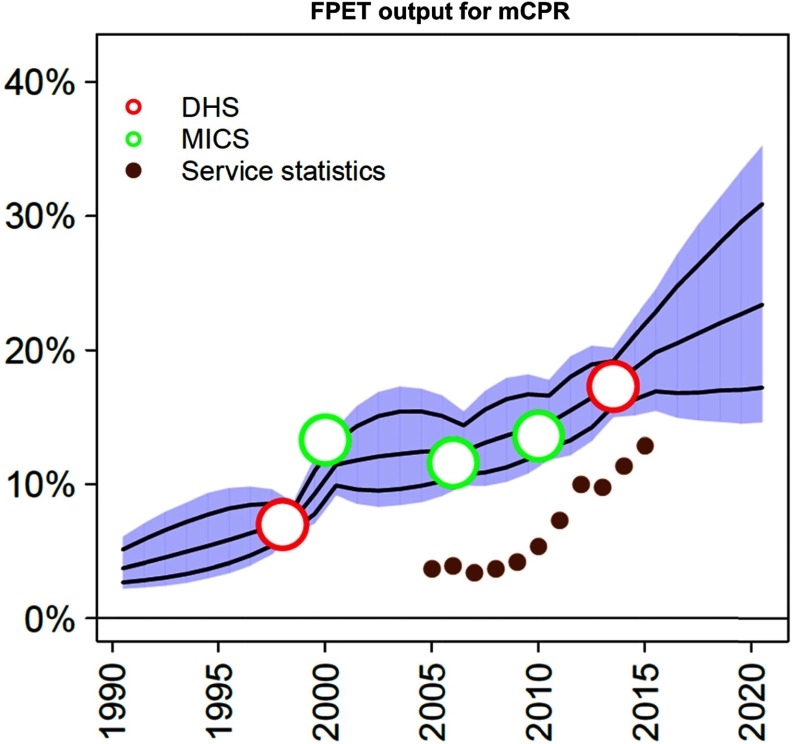
Using Service Statistics in FPET to Inform Trends in mCPR Growth Abbreviations: DHS, Demographic and Health Surveys; FPET, Family Planning Estimation Tool; MICS, Multiple Indicator Cluster Surveys; mCPR, modern contraceptive prevalence rate.

**FPET combines survey data and service statistics to inform trends in mCPR growth:** Track20's FPET,^15^ adapted from a model used by the United Nations Population Division,^16^ generates statistical estimates of mCPR that are informed by survey data as well as regional and global patterns of change. FPET has been modified to allow service statistics (either client visits by method or commodities distributed to clients by method) from government HMISs to inform the trajectory of mCPR growth after the latest survey. These service statistics are converted into an Estimated Modern Use (EMU). This value is not directly comparable with the mCPR (due to limitations and biases within routine data); however, the shape of the trend is used to inform the progress of mCPR growth. For example, in Figure 2 service statistics are seen to be trending well with surveys for a number of years and can now be used to inform annual progress after the 2013–2014 DHS survey. This allows service statistics to inform projections of mCPR (and therefore of additional users) indirectly, circumventing the issues discussed above that do not allow HMIS data to be directly extrapolated to estimates of national-level mCPR changes.

**FIGURE 3:**
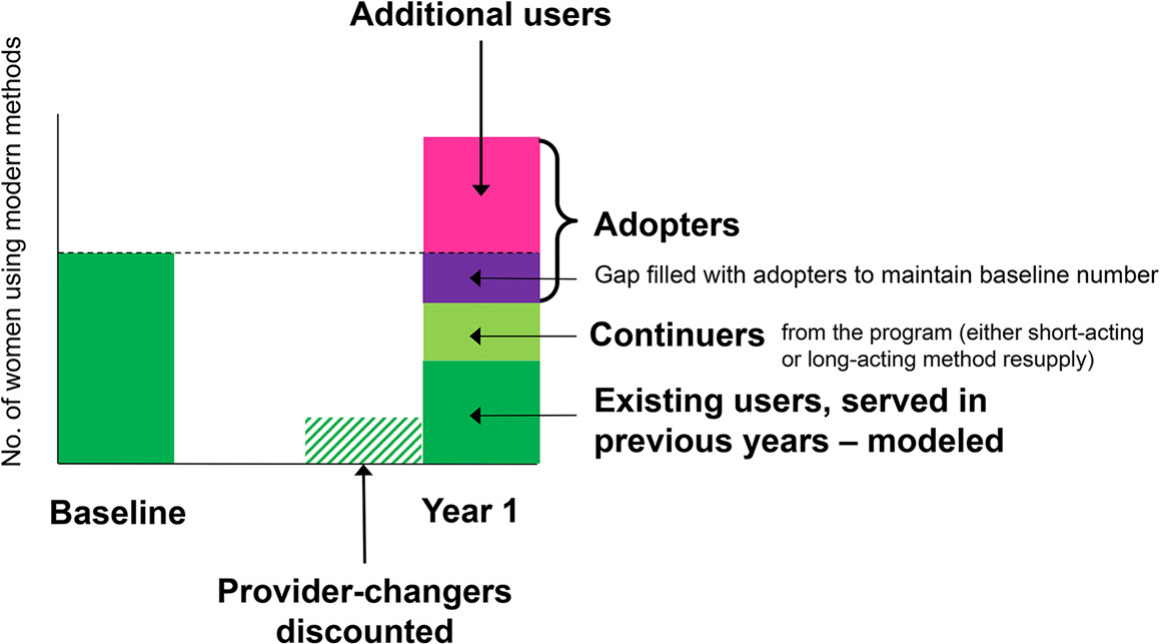
How Contribution to Additional Users Are Modeled in Impact 2 Source: Adapted from Weinberger, Fry, and Hopkins (2015).^18^

**Impact 2 model uses service statistics and client-use profile data to estimate contributions to additional users:** The Impact 2 model, developed by Marie Stopes International, allows organizations to estimate their contribution to national-level additional users, based on program-level and other input data.^17^^,^^18^ First, the number of services provided is converted into the number of estimated users in a given country (accounting for long-acting and permanent method continuation, mortality, and short-acting methods needed for a year of coverage). Next, client-use profile data (the proportion that are adopters, provider-continuers, and provider-changers; see the Box) are used to allocate users to 1 of 3 categories: (1) any growth in users that came from provider-changers are discounted (“substitution effect”);^19^ (2) the previous year's baseline must be maintained with provider-continuers and adopters; and (3) only the remaining adopters are allowed to contribute to further growth. This approach uses the client-use profile information to model how the organizations' increases in users are likely changing national contraceptive use levels. Rather than directly counting increases in users or relying wholly on program-level indicators such as first-time users or adopters, the model accounts for the full dynamics of continuation, discontinuation, and substitution between providers in order to estimate contribution to population-level change (Figure 3). This approach is comparable with FP2020's concept of additional users.

For more information about FPET and the Impact 2 model, see the supplement.

## FINAL REFLECTIONS

We call on government ministries, service providers, and donors to align how they define and track their contributions to FP2020, so that across the sector we are all aiming for the same goal and measuring the same concept. Doing so will strengthen the field's ability to respond to important measurement challenges as we transition from the FP2020 goals to those of universal access to reproductive health and the 2030 Sustainable Development Goals that follow.

There is much focus on the goal of ensuring that an additional 120 million women use contraception by 2020. What has been less visible from this goal is the 270 million women in the world's poorest 69 countries who were estimated to be already using contraception in 2012.^8^ This number of users will need to be maintained before any progress can be made toward reaching the additional 120 million women. Maintaining this base takes work; those who rely on short-acting methods will need continual resupply and those using long-acting methods may need their methods replaced. In addition, some women will drop out of contraceptive use, either because they will age beyond their reproductive years or discontinue either due to method-related problems or because they no longer have a need for contraception. Therefore, there will always be a need to reach adopters as part of the efforts to sustain existing contraceptive use in any given population. Only when looking at the complete picture can we see to what degree our efforts are resulting in actual increases in modern contraceptive users at the national and global levels.

Service-level outputs (services provided, client visits, and CYPs) play an important role in monitoring family planning programs, and thus their tracking should not be undervalued. As HMISs improve with technological advances in electronic data collection and analysis, enabling more robust collection of longitudinal metrics that track individual clients' first-time use, discontinuation, method switching, resumption of use, and so on, even more value can be obtained from routine tracking. However, due to the limitations described in this article, these program-level measures will never be directly comparable with population-level changes in contraceptive use such as additional users.

Finding ways to bridge the gap between program and population measures is important. Using validated models allows implementing organizations, governments, and donors to translate service statistics into meaningful estimates of national-level changes in continued contraceptive use. For example, from experience in the social franchising space, the Metrics Working Group has defined the metric of choice for measuring “additionality” in contraceptive use to be the “contribution to additional users as modeled by the Impact 2 model.”^20^ The group recognized the importance of not only measuring the scale of family planning provider networks (through metrics such as CYPs) but also going the next step to understand how the provider network is contributing to national-level changes in contraceptive use.

We have outlined how national-level estimates of additional users can be informed by routine data and how individual organizations can estimate their contribution to additional users. Therefore, we call on donor agencies, governments, implementing organizations, and other partners to:

- Focus on “additional users” as an indication of sustained growth in contraceptive use at the national level
- Drop “new users” and “acceptors” from the family planning measurement agenda as these ambiguous terms create confusion
- Focus on capturing data on “adopters” as the preferable client characteristic to better understand who is being reached by family planning programs and to inform modeled estimates of additional users
- Use output measures such as client visits, services provided, and CYPs not only to inform program monitoring but also to inform modeled estimates of additional users and to better understand the link between the service and population levels

Collectively, these steps will ensure that across the family planning sector we are using a standardized and comparable terminology and approach to define and measure progress. More importantly, they will ensure that growth is measured in a way that takes into account both growth beyond a baseline level of use and provision of services to women who were not previously protected by contraceptives. If the additionality metric is widely adopted, programs will be able to immediately see their contributions to the global goal of enabling women to access modern contraception.

## Supplementary Material

Supplemental material

## Notes

**Peer Reviewed**
