# Supplementary material for: “New Users” Are Confusing Our Counting: Reaching Consensus on How to Measure “Additional Users” of Family Planning
Source: Glob Health Sci Pract. 2017 Mar 24;5(1):6–14. doi: 10.9745/GHSP-D-16-00328 (PMC5478230; doi:10.9745/GHSP-D-16-00328)
Supplement: Supplemental material [file supp_5_1_6__index.html]

“New Users” Are Confusing Our Counting: Reaching Consensus on How to Measure “Additional Users” of Family Planning — Supplemental material 

# “New Users” Are Confusing Our Counting: Reaching Consensus on How to Measure “Additional Users” of Family Planning

## Supplemental material

- Text s01, PDF - Text s01, PDF
