# Supplementary material for: “New Users” Are Confusing Our Counting: Reaching Consensus on How to Measure “Additional Users” of Family Planning
Source: Glob Health Sci Pract. 2017 Mar 24;5(1):6–14. doi: 10.9745/GHSP-D-16-00328 (PMC5478230; doi:10.9745/GHSP-D-16-00328)
Supplement: Supplemental material [file GHSP-D-16-00328_16-00328-Dasgupta-Supplement.pdf]

Dasgupta A, Weinberger M, Bellows B, Brown W. “New users” are confusing our counting: reaching consensus on how to measure “additional users” of family planning. *Glob Health Sci Pract*. 2017;5(1).
http://dx.doi.org/10.9745/GHSP-D-16-00328

Supplement: Tools and Models to Bridge the Gap Between Service-Level Measures Available in a Program’s Routine Service Statistics and Population-Level Metrics

Track20’s Family Planning Estimation Tool

Track20 was established to track progress of the global Family Planning 2020 (FP2020) initiative, support national efforts to improve family planning monitoring, and use this monitoring to inform effective program strategies and plans. Central to Track20’s work is developing and improving methodologies that allow for tracking progress on an annual basis. Historically most countries have had to rely on tracking increases in the modern contraceptive prevalence rate (mCPR) through population-based surveys that are conducted only on a periodic basis. The Family Planning Estimation Tool (FPET), adapted from a model used by the United Nations Population Division,[1] allows for estimates of mCPR to be informed by historic survey data, services statistics, and regional and global patterns of change.

The introduction of service statistics into FPET has provided a new way for routine data to be used to inform estimates of “additional users.” Generally, 3 types of service statistics can be used in FPET: (1) client visit data (by method), (2) data on commodities distributed to clients, or (3) less preferable, data on commodities distributed to facilities. A series of validation checks are performed to ensure that the trend seen in the service statistics represents actual changes in the coverage of services (rather than, for example, increases in reporting rates). Further, because we know that service statistics are not directly comparable with mCPR for many of the reasons discussed in this supplement’s referring article, FPET treats these data differently than data from population-based surveys. Namely, FPET is informed by the shape of the trend created from service statistics, but it is not influenced by the level. This recognizes that while there may be biases in service statistics data, if the biases are consistent, the shape of the trend can still be informative.

Track20 has developed a tool to allow countries to convert their services statistics into Estimated Modern Use (EMU), a term developed for the modeled prevalence from service statistics that recognizes that this prevalence is not directly comparable with mCPR. The tool includes data validation mechanisms to help countries decide if their data are fit for inclusion in FPET. If they are, the EMU trend is added to the FPET and informs the mCPR trajectory. This mCPR trajectory, coupled with data on women of reproductive age, is then used to estimate additional users in a given year. This process allows service statistics to *inform* the additional users estimate indirectly, circumventing the issues that arise when individual-level routine data cannot account for the full dynamics of contraceptive use.

FPET can be accessed at http://fpet.track20.org/.

Marie Stope International’s Impact 2 Model

The FPET solution is focused on estimating national changes in contraceptive use. However, in many cases organizations or partners want to estimate their *contribution* to “additional users” as a way to understanding their impact and as part of organizational advocacy. However, in a country with multiple service providers and organizations, estimating individual contributions to the FP2020 goal of “120 million additional users” is not straightforward for 2 reasons:

1. Service providers typically collect data on services and commodities provided, not the number of family planning users.
2. The issue of substitution must be addressed—an increase in users might not translate into a national increase if they were drawn from a pool of existing users previously served by another provider.

Marie Stopes International’s Impact 2 model is designed to address both of these issues. Service providers generally rely on data about family planning services provided or commodities distributed. This is not directly equivalent to contraceptive use in a given year since (1) long-acting and permanent methods (LAPMs) are used for multiple years, and (2) each short-acting method (e.g., each condom distributed or injection provided) does not equate to 1 contraceptive user in a given year. To address this, the Impact 2 model estimates the number of modern contraceptive users based on services provided in the reference year, as well as LAPMs provided in previous years with discontinuation and mortality rates applied. Continuation of LAPMs is built into the model, so there are estimates of users continuing to use their LAPM from year to year. Further, the model converts short-acting methods provided into users, based on the average number of commodities needed for a year of coverage.

Once user numbers have been estimated, it would not be appropriate to simply calculate the difference in the absolute number of users at endline compared with baseline. This is because some of the growth in users from baseline to endline could be due to service provision to clients who have switched service providers (the “substitution effect”).[15] To address this issue, the Impact 2 model can be used with client-use profile data (adopters, provider-continuers, and provider-changers; see the Box in this supplement’s referring article) to model the contribution to increasing national contraceptive use (either in absolute terms as “additional users” or relative terms as mCPR).

A simplified version of this calculation is shown in Figure 3 in this supplement’s referring article. First, the model accounts for continuation of use among LAPM clients served in previous years (dark green). Then, Impact 2 models out additional users by accounting for services that are provided in subsequent years. Provider-continuers (clients from the program who need resupply or are switching methods) are reflected in light green. In order to maintain the baseline number of women provided with family planning, the gap caused by discontinuation or women choosing to go to a different provider must be filled by adopters (shown in purple). Only once that gap has been filled can adopters served on top of that be counted as additional users (shown in pink).

No provider-changers contribute to additional users and thus are indicated in striped green next to the Year 1 stack in Figure 3, since this growth would be considered substitution, rather than contributing to market increases. This approach is in line with FP2020’s measurement of additional users. We highlight the fact that this approach requires client-use profile data and recommend that organizations value the importance of collecting these data, either through surveys or through routine data sources, to understand who their clients are and estimate contribution to additional users.

Although it is more complex to model an organization’s contribution to additional users than to simply track program outputs (e.g., services provided or couple-years of protection [CYPs]) or even client-profile information (e.g., proportion of adopters or first-time users), we believe that this is needed to adequately track national-level contributions of individual providers, family planning organizations, and donors. Modeling these outcomes ensures that the complete picture of sustaining and increasing use is taken into consideration. The Impact 2 approach has recently been endorsed by the Social Franchising Metrics Working Group as the metric of choice for measuring “additionality” in family planning.[2] The group recognized the importance of not only measuring the scale of social franchises (through metrics like CYPs) but also going the next step to understand how the social franchise network is contributing to national-level changes in contraceptive use.

Impact 2 can be downloaded at https://mariestopes.org/what-we-do/our-approach/our-technical-expertise/impact-2/.

References

1. Alkema L, Kantorova V, Menozzi C, Biddlecom A. National, regional, and global rates and trends in contraceptive prevalence and unmet need for family planning between 1990 and 2015: a systematic and comprehensive analysis. *Lancet*. 2013;381(9878):1642-1652. CrossRef. Medline
2. Social Franchise Metrics Working Group (SFMWG). Additionality: capturing additional users in a health market. SFMWG; 2016. http://www.sf4health.org/sites/sf4health.org/files/wysiwyg/additionality5.pdf. Accessed August 26, 2016.
